# Supplementary material for: Single-cell sequencing of immune cells from anticitrullinated peptide antibody positive and negative rheumatoid arthritis
Source: Nat Commun. 2021 Aug 17;12:4977. doi: 10.1038/s41467-021-25246-7 (PMC8371160; doi:10.1038/s41467-021-25246-7)
Supplement: Supplementary file 1 — Supplementary Information [file 41467_2021_25246_MOESM1_ESM.pdf]

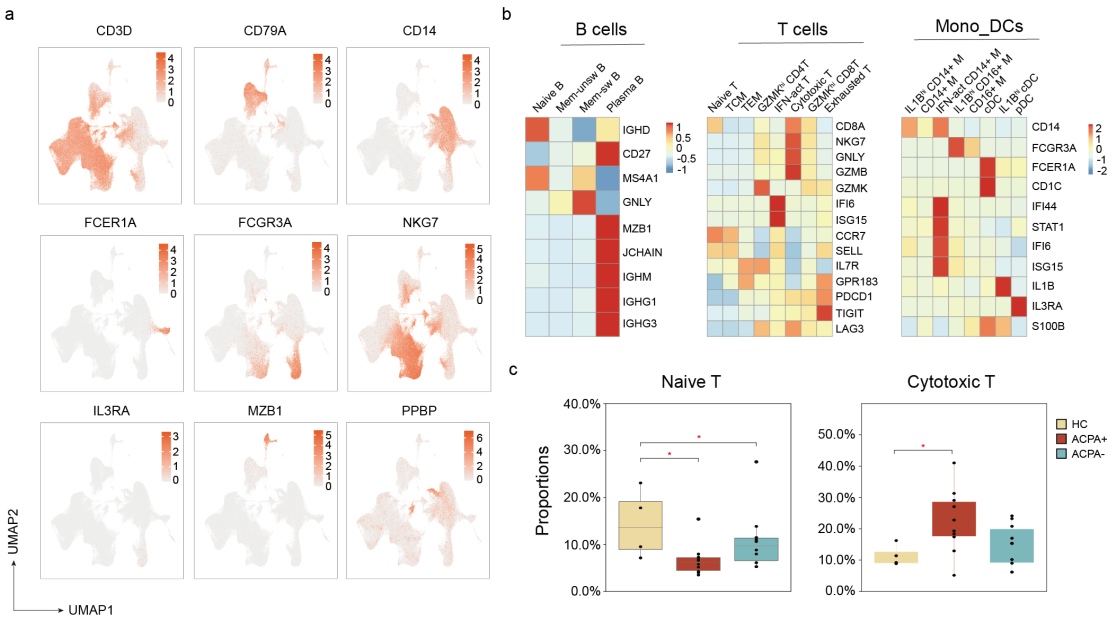

Supplementary Figure 1

Supplementary Figure 1. Gene expressions of immune cells subsets in PBMCs of RA patients

a. UMAP plots showing the expression of marker genes in major cell types in PBMC. Cells are color-coded by their log-normalized counts.

b. Z-score normalized mean expression of selected cell function-associated genes in each cell cluster in PBMC, separated by major cell types (B cell, T/NK cells and myeloid cells).

c. Cell clusters showed enrichment in particular ACPA groups. P values were calculated using a two-sided unpaired Wilcoxon test. *$p<0.05$ (from left to right, p = 0.024, 0.043,0.036). n=4 for HC, n=10 for ACPA+ group, and n=10 for ACPA- group.

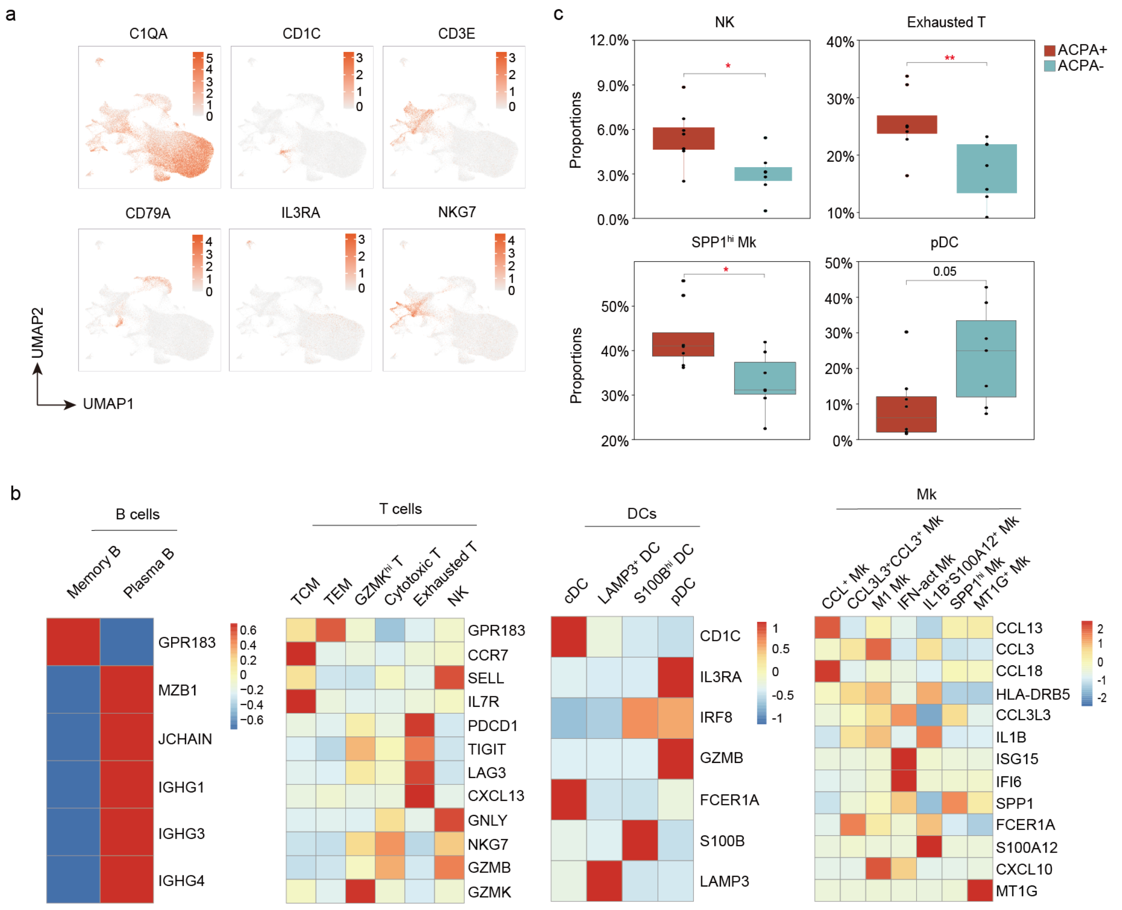

Supplementary Figure 2

Supplementary Figure 2. Gene expressions of immune cells subsets in synovium of RA patients

A. UMAP plots showing the expression of marker genes in major cell types in STMC. Cells are color-coded by their log-normalized counts.

B. Z-score normalized mean expression of selected cell function-associated genes in each cell cluster in STMC, separated by major cell types (B cell, T cells, dendritic cells, and macrophages).

C. Cell clusters showed enrichment in particular ACPA groups. P values were calculated using a two-sided unpaired Wilcoxon test. **p<0.01 (p = 0.0059), *p<0.05 (from top to bottom, p = 0.021,0.029). n=10 for ACPA+ group, and n=10 for ACPA- group.

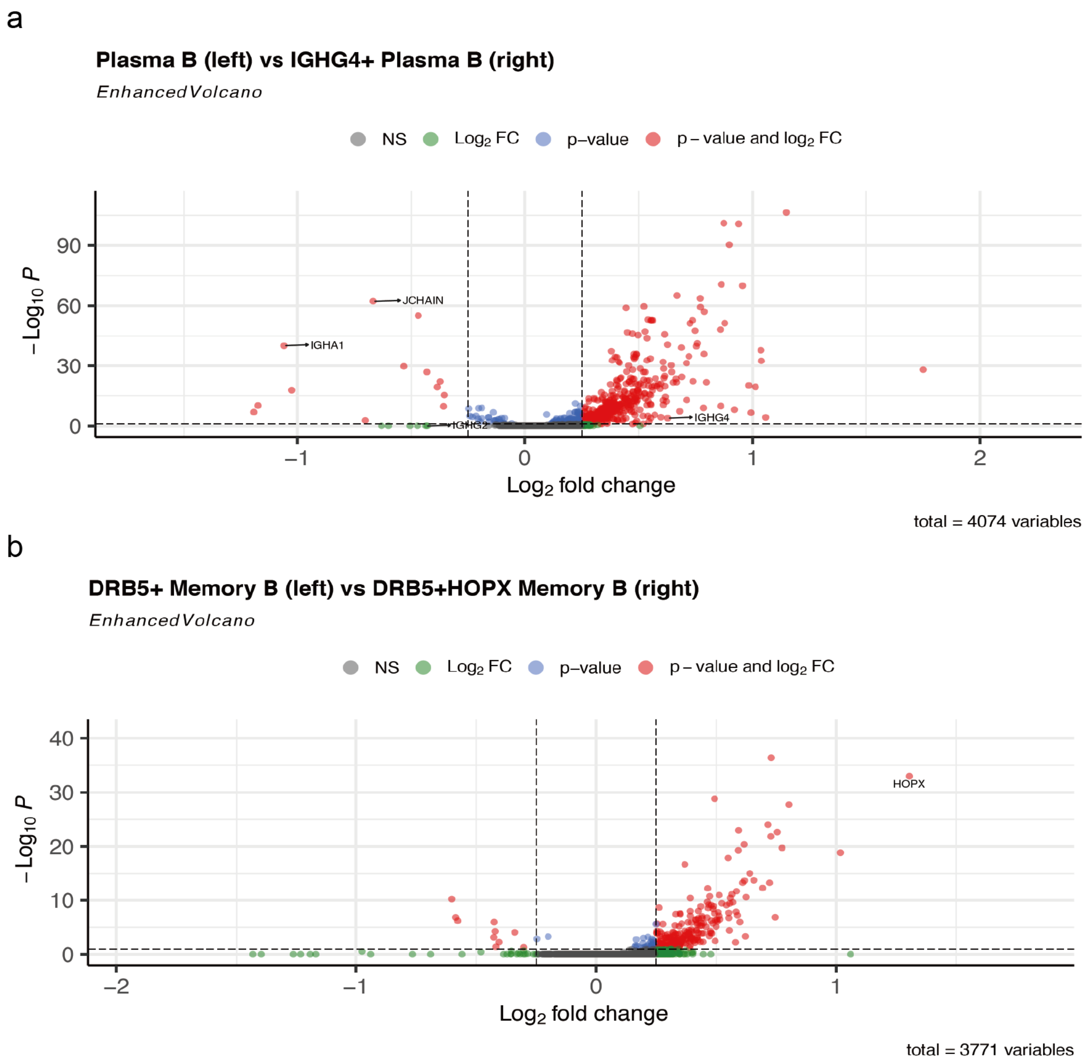

Supplementary Figure 3

Supplementary Figure 3. Differential gene expression in different B cells subsets.

A. Volcano plots showing differential expressed genes (DEGs) of IGHG4^{+} Plasma B cells in comparison with Plasma B cells. Red dots indicate genes with a P value < 0.05 and a $\log_2$(fold change) > 0.5. Concerned DEGs are marked in the plots. DEGs were identified using two-sided Wicoxon Rank Sum test, and p value were adjusted based on Bonferroni correction using all expressed genes in the dataset.

B. Volcano plot showing DEGs of HLA-DRB5^{+}HOPX^{+} memory B compared with HLA-DRB5^{+} memory B. Red dots indicate genes with a P value < 0.05 and a $\log_2$(fold change) > 0.5. Concerned DEGs are marked in the plots. DEGs were identified using two-sided Wicoxon Rank Sum test, and p value were adjusted based on Bonferroni correction using all expressed genes in the dataset.

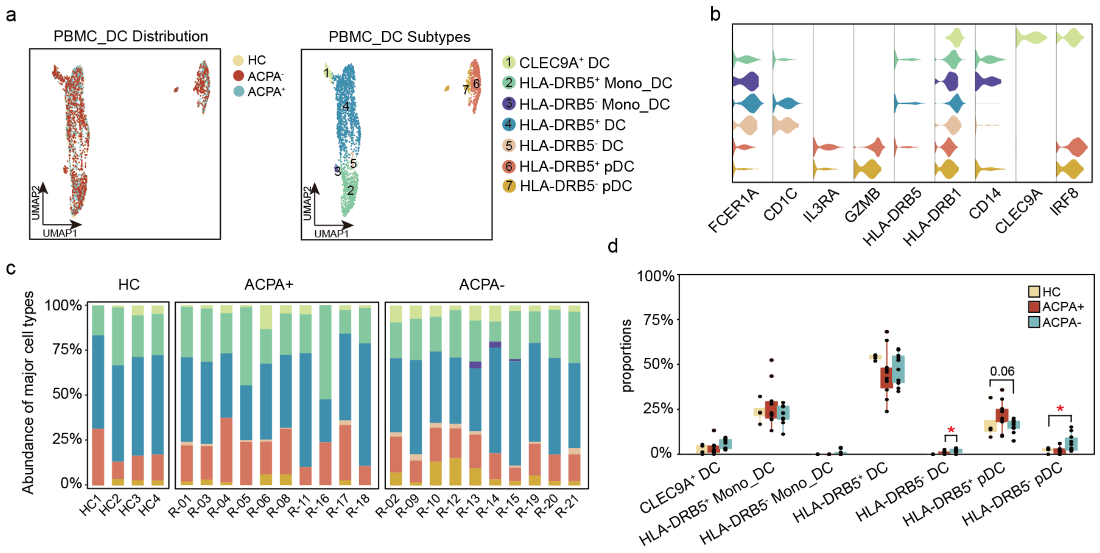

Supplementary Figure 4

Supplementary Figure 4. Classification of dendritic cells in PBMC

A. UMAP visualization of dendritic cells from PBMC, with 7 cell subtypes identified across 2,902 cells. Cells are marked by ACPA type(left) and cell subtypes (right).

B. Violin plots showing marker genes across PBMC DC subtypes in A. The y axis represents log-scaled normalized counts.

C. Bar plots showing the relative percentage of DC subtypes in PBMC for each sample.

D. Box plots showing the proportions of each DC subtype in PBMC across ACPA groups. Cell types showed enrichment in ACPA+ or ACPA- subgroups are marked with *. P values were calculated by the two-sided Wilcoxon test. *$p<0.05$ ($p = 0.031$). n=4 for HC, n=10 for ACPA+ group, and n=10 for ACPA- group.

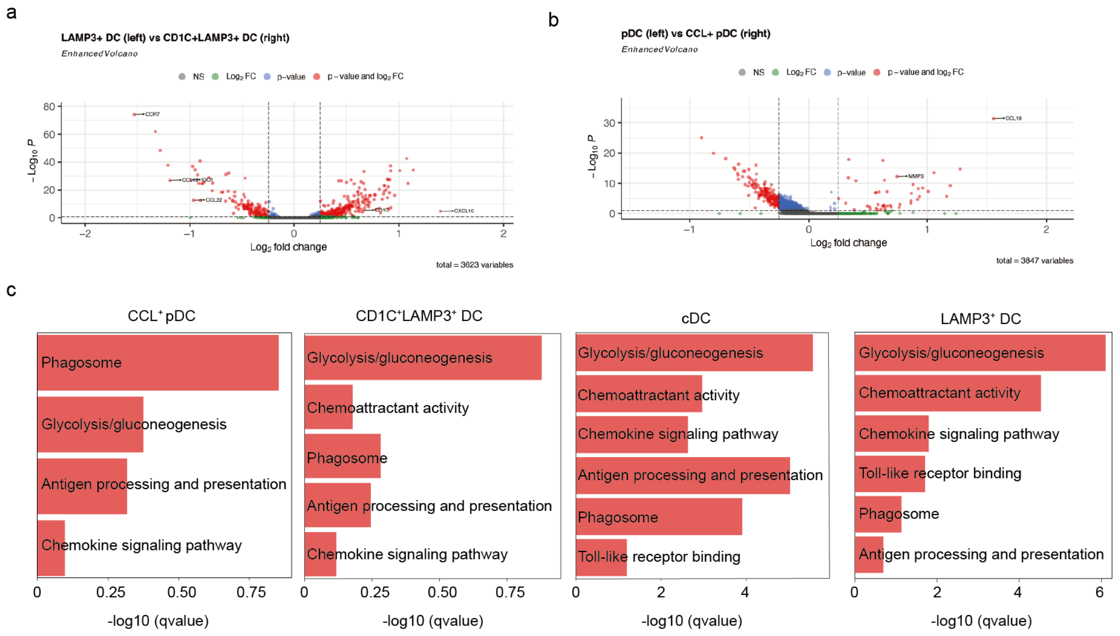

Supplementary Figure 5

Supplementary Figure 5. Differential gene expression in PBMC DC subsets.

A. Volcano plots showing the differentially expressed genes (DEGs) between LAMP3+ DC and CD1C+LAMP3+ DC. Red dots indicate genes with a P value < 0.05 and a $\log_2$(fold change) > 0.5. Concerned DEGs are marked in the plots. DEGs were identified using two-sided Wicoxon Rank Sum test, and p value were adjusted based on Bonferroni correction using all expressed genes in the dataset.

B. Volcano plots showing the differentially expressed genes (DEGs) between pDC and CCL+ pDC. Red dots indicate genes with a P value < 0.05 and a $\log_2$(fold change) > 0.5. Concerned DEGs are marked in the plots. DEGs were identified using two-sided Wicoxon Rank Sum test, and p value were adjusted based on Bonferroni correction using all expressed genes in the dataset.

C. Bar plots showing the enriched upregulated KEGG pathways and GO biological processes terms of specific DC cell subtypes.

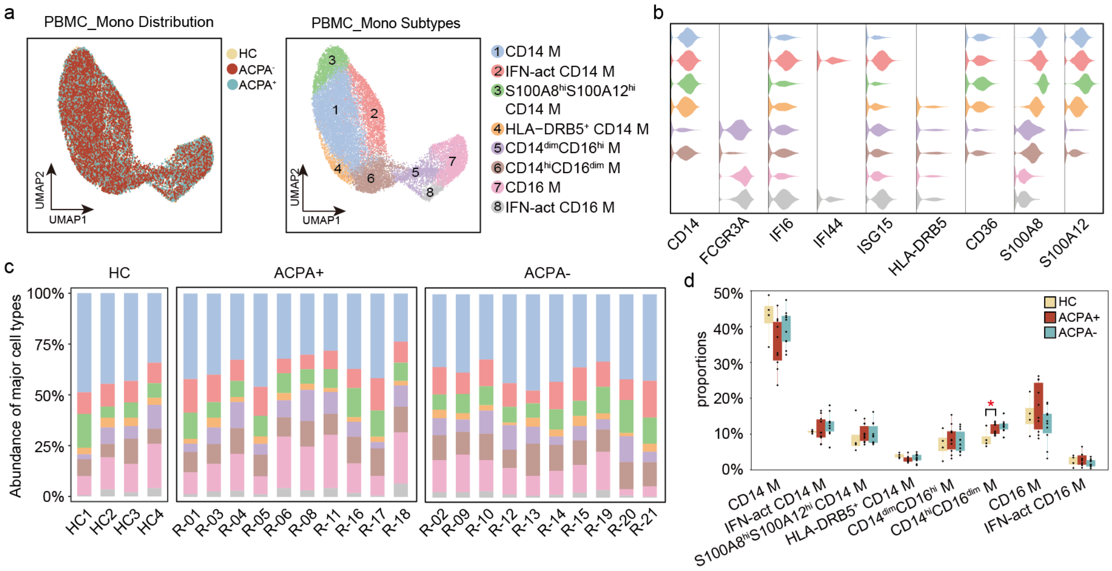

Supplementary Figure 6

Supplementary Figure 6. Classification of monocytes in PBMC.

A. UMAP visualization of monocytes from PBMC, with 8 cell subtypes identified across 24,661 cells. Cells are marked by ACPA type(left) and cell subtypes (right).

B. Violin plots showing marker genes across PBMC monocyte subtypes in A. The y axis represents log-scaled normalized counts.

C. Bar plots showing the relative percentage of monocyte subtypes in PBMC for each sample.

D. Box plots showing the proportions of each monocyte subtype in PBMC across ACPA groups. Cell types showed enrichment in ACPA+ or ACPA- subgroups are marked with *. P values were calculated by the two-sided Wilcoxon test. *$p<0.05$ ($p = 0.036$). n=4 for HC, n=10 for ACPA+ group, and n=10 for ACPA- group.

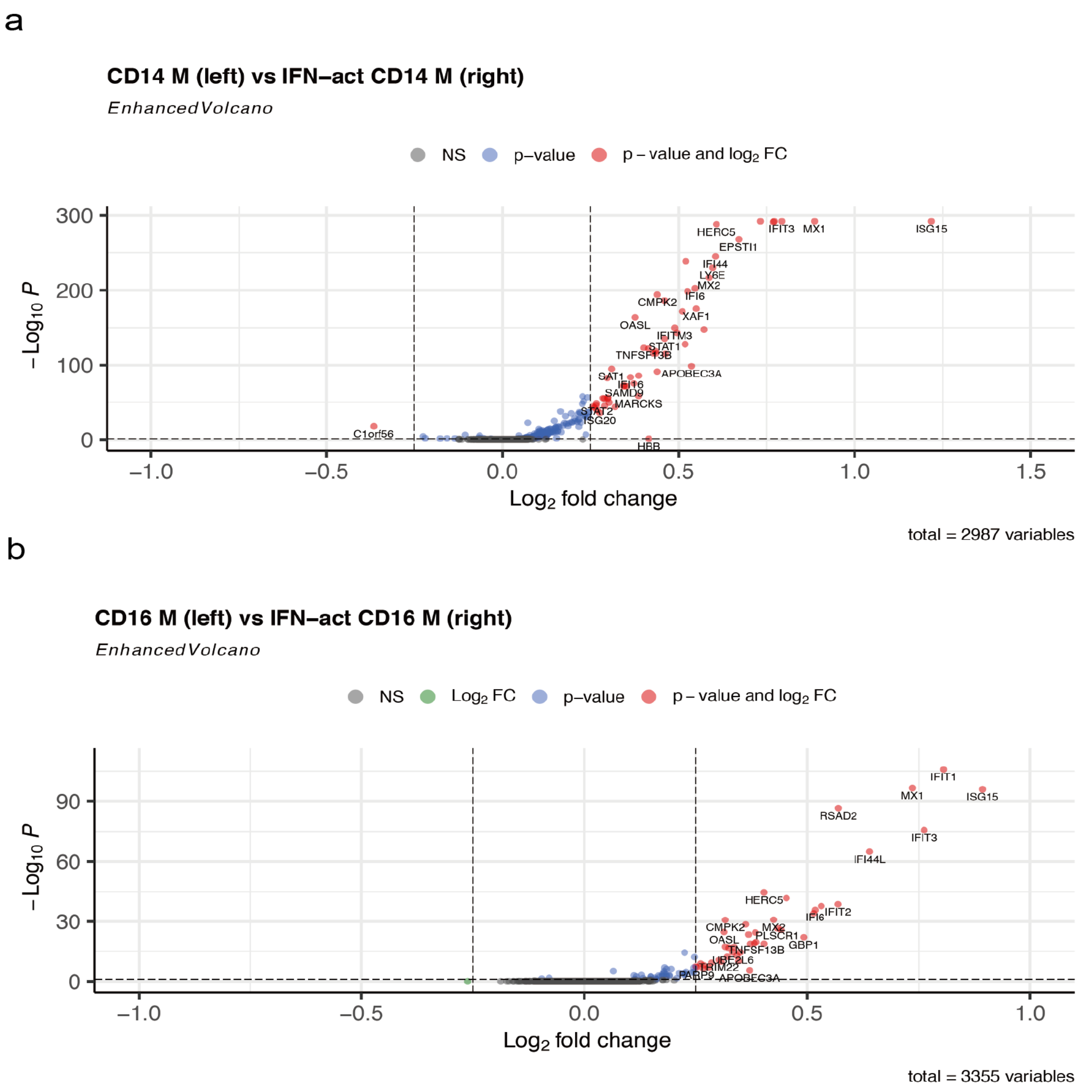

Supplementary Figure 7

Supplementary Figure 7. Differential gene expression in PBMC monocyte subsets.

A. Volcano plots showing the differentially expressed genes (DEGs) between CD14+ M and IFN-act CD14+ M. Red dots indicate genes with a P value < 0.05 and a $\log_2$(fold change) > 0.5. Concerned DEGs are marked in the plots. DEGs were identified using two-sided Wicoxon Rank Sum test, and p value were adjusted based on Bonferroni correction using all expressed genes in the dataset.

B. Volcano plots showing the DEGs between CD16+ M and IFN-act CD16+ M. Red dots indicate genes with a P value < 0.05 and a $\log_2$(fold change) > 0.5. Concerned DEGs are marked in the plots. DEGs were identified using two-sided Wicoxon Rank Sum test, and p value were adjusted based on Bonferroni correction using all expressed genes in the dataset.

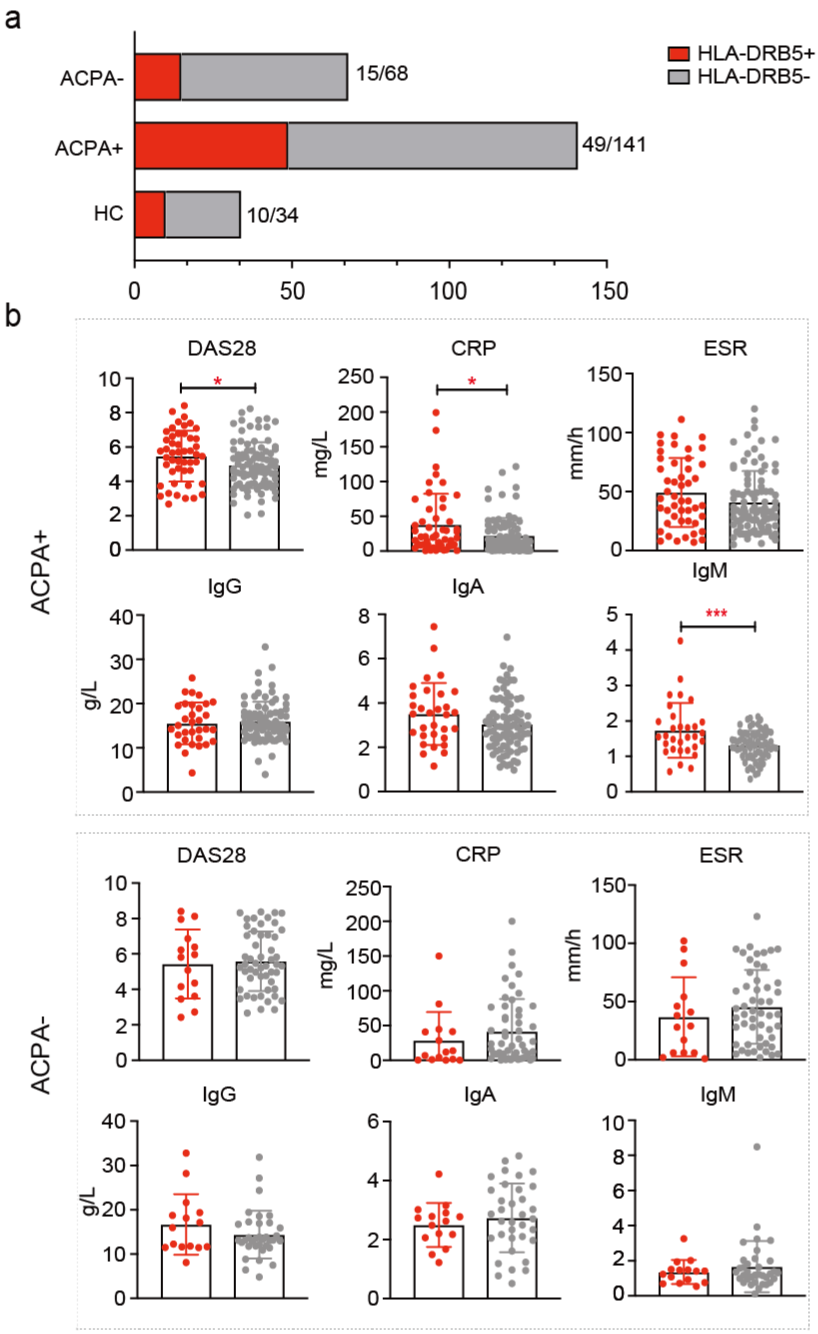

Supplementary Figure 8

Supplementary Figure 8. *HLA-DRB5* Genotyping for CCL3, CCL13, and CCL18

A. Frequency of the HLA-DRB5 genotype in 34 healthy controls, 141 ACPA+, and 68 ACPA- RA patients.

B. Comparison of DAS (Disease Activity Score) 28, CRP (C-reactive Protein), ESR (Erythrocyte Sedimentation Rate), IgG, IgA, and IgM levels in HLA-DRB5 + and - patients in different RA subgroups. Error bars show mean ± S.D. P values were calculated by the two-sided Mann-Whitney test. *p<0.05 (from left to right, p = 0.033, p = 0.039), ***p<0.001 (p =0.0006). In ACPA+ group, n = 49 for HLA-DRB5+ group and n = 92 for HLA-DRB5- group; in ACPA- group, n = 15 for HLA-DRB5+ group and n=35 for HLA-DRB5- group.

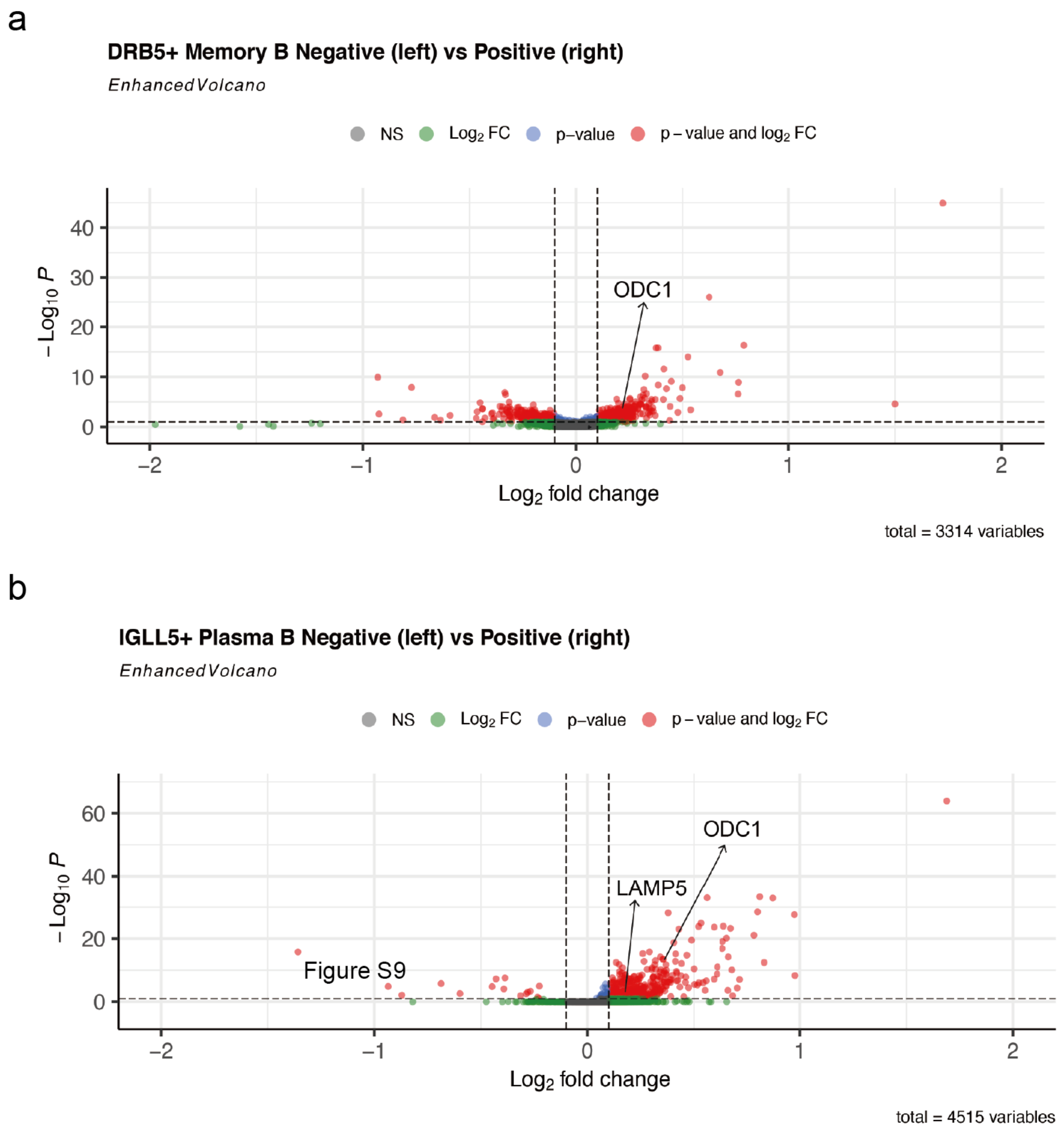

Supplementary Figure 9

Supplementary Figure 9. Differential gene expression in specific synovial B cell subsets between ACPA+ and ACPA- RA patients.

A. Volcano plots showing the differentially expressed genes (DEGs) in HLA-DRB5+ Memory B cells between ACPA+ and ACPA- RA patients. Red dots indicate genes with a P value < 0.05 and a log_2(fold change) > 0.5. Concerned DEGs are marked in the plots. DEGs were identified using two-sided Wicoxon Rank Sum test, and p value were adjusted based on Bonferroni correction using all expressed genes in the dataset.

B. Volcano plots showing the differentially expressed genes (DEGs) in IGLL5+ Plasma B cells between ACPA+ and ACPA- RA patients. Red dots indicate genes with a P value < 0.05 and a log_2(fold change) > 0.5. Concerned DEGs are marked in the plots. DEGs were identified using two-sided Wicoxon Rank Sum test, and p value were adjusted based on Bonferroni correction using all expressed genes in the dataset.

Supplementary Table 1. Patient characteristics for single cell samples

Characteristics	ACPA+ (n=10)	ACPA- (n=10)
**Female**	10	10
**Age (years)**	53.6 (20~77)	43.8 (28~56)
**CCP-positive**	10	0
**RF-positive**	10	1
**Duration (years)**	7.7 (1m ~ 18y)	5.1 (1m ~ 18y)
**DAS28**	5.45 ± 0.96	5.51 ± 1.68

Supplementary Table 2. Patient Characteristics for HLA-DRB5 Genotyping

Characteristics	ACPA+ (N = 141)		ACPA- (N = 68)	
	HLA-DRB5+ (n = 49)	HLA-DRB5- (n = 92)	HLA-DRB5+ (n = 15)	HLA-DRB5- (n = 53)
**Female**	43	77	12	40
**Age (year)**	49.3 ± 13.4	48.0 ± 13.6	48.7 ± 13.0	50.5 ± 11.7
**DAS28**	5.47 ± 1.49*	4.94 ± 1.34	5.43 ± 1.95	5.59 ± 1.68
**ESR (mm/h)**	49.21 ± 29.23	41.10 ± 26.19	36.93 ± 33.85	45.46 ± 31.71
**CRP (mg/L)**	37.88 (0.51~199.2)*	22.17 (0.5~121.6)	28.56 (0.22~150.1)	41.86 (0.57~200.0)
**IgG (g/L)**	15.53 ± 4.72	15.99 ± 4.44	16.67 ± 6.83	14.36 ± 5.40
**IgA (g/L)**	3.51 ± 1.40	3.04 ± 1.21	2.50 ± 0.74	2.74 ± 1.17
**IgM (g/L)**	1.74 ± 0.78***	1.32 ± 0.41	1.36 ± 0.69	1.67 ± 1.46

Data are shown as mean ± S.D. P values were calculated by the two-sided Mann-Whitney test. *p<0.05, **p<0.01, ***p<0.001.

Supplementary Table 3. Summary of scRNA-seq of ACPA- RA as compared to ACPA+ RA patients

Subsets	Synovial Tissue	Peripheral Blood
**B cells**	① *HLA-DRB5* expression↓ in memory B cells;<br>② "antigen processing and presentation activity" ↓.	① *IGHG4*$^{+}$ plasma B↑;<br>② *HLA-DRB5*$^{+}$ Plasma B↓.
**Dendritic Cells**	① *CCL13*, *CCL18* and *MMP3*↑;<br>② "phagosome", "glycolysis/gluconeogenesis" and "antigen processing and presentation activity" ↓.	① *HLA-DRB5*$^{-}$ pDC and DC↑;<br>② *HLA-DRB5*$^{+}$ pDC↓.
**Monocyte/ Macrophage**	① *CCL13*, *CCL18* and *MMP3*↑;<br>② *HLA-DRB5* expression↓;<br>③ *IL1B* gene expression↑.	No obvious differences
**T cells**	① *NK* cells and exhausted CD4 T cells↓;<br>② cytotoxic and exhausted gene expression↓;<br>③ Distinct high expression of *MMP3*.	① *S100A8*^{hi}*GZMB*$^{+}$ Teff cells↑;<br>② *GZMK*$^{+}$ IFN-act TCM↑
