# Supplementary material for: Single-cell sequencing of immune cells from anticitrullinated peptide antibody positive and negative rheumatoid arthritis
Source: Nat Commun. 2021 Aug 17;12:4977. doi: 10.1038/s41467-021-25246-7 (PMC8371160; doi:10.1038/s41467-021-25246-7)
Supplement: Supplementary file 3 — Reporting Summary [file 41467_2021_25246_MOESM3_ESM.pdf]

nature research

Corresponding author(s): George C. Tsokos; Fan Bai; Xuan Zhang

Last updated by author(s): Jul 11, 2021

Reporting Summary

Nature Research wishes to improve the reproducibility of the work that we publish. This form provides structure for consistency and transparency in reporting. For further information on Nature Research policies, see our Editorial Policies and the Editorial Policy Checklist.

Statistics

For all statistical analyses, confirm that the following items are present in the figure legend, table legend, main text, or Methods section.

n/a	Confirmed	
☐	☒	The exact sample size (*n*) for each experimental group/condition, given as a discrete number and unit of measurement
☐	☒	A statement on whether measurements were taken from distinct samples or whether the same sample was measured repeatedly
☐	☒	The statistical test(s) used AND whether they are one- or two-sided<br>*Only common tests should be described solely by name; describe more complex techniques in the Methods section.*
☒	☐	A description of all covariates tested
☐	☒	A description of any assumptions or corrections, such as tests of normality and adjustment for multiple comparisons
☒	☐	A full description of the statistical parameters including central tendency (e.g. means) or other basic estimates (e.g. regression coefficient) AND variation (e.g. standard deviation) or associated estimates of uncertainty (e.g. confidence intervals)
☐	☒	For null hypothesis testing, the test statistic (e.g. *F*, *t*, *r*) with confidence intervals, effect sizes, degrees of freedom and *P* value noted<br>*Give P values as exact values whenever suitable.*
☒	☐	For Bayesian analysis, information on the choice of priors and Markov chain Monte Carlo settings
☒	☐	For hierarchical and complex designs, identification of the appropriate level for tests and full reporting of outcomes
☒	☐	Estimates of effect sizes (e.g. Cohen's *d*, Pearson's *r*), indicating how they were calculated

Our web collection on statistics for biologists contains articles on many of the points above.

Software and code

Policy information about availability of computer code

Data collection

Raw 10x Genomics sequencing data were processed using the CellRanger software v. 2.2.0, and the 10x human transcriptome GRCh38-1.2.0 was used as the reference.

Data analysis

Analysis of single cell RNA-seq data was done using R (v3.6.1) with publicly available packages. Dimensionality reduction and differential gene expression was performed using the Seurat (v3.1.1) package. Plots were generated using the ggplot2 (v 3.2.1), pheatmap (v 1.0.12), and EnhancedVolcano (v 1.2.0) packages. Gene ontology analysis was performed using the clusterProfiler (v3.12.0) package and the DAVID 6.8 web resource. Trajectory analysis was conducted using the Monocle 3 package. Cell-cell interaction prediction was done using CellPhoneDB (v2.1.2). DRB1-DRB5 haplotypes were determined by BGI (Beijing Genomics Institute) using the SS-SBT method.

For manuscripts utilizing custom algorithms or software that are central to the research but not yet described in published literature, software must be made available to editors and reviewers. We strongly encourage code deposition in a community repository (e.g. GitHub). See the Nature Research guidelines for submitting code & software for further information.

Data

Policy information about availability of data

All manuscripts must include a data availability statement. This statement should provide the following information, where applicable:

- Accession codes, unique identifiers, or web links for publicly available datasets
- A list of figures that have associated raw data
- A description of any restrictions on data availability

The raw sequence data reported in this paper have been deposited in the Genome Sequence Archive in BIG Data Center, Beijing Institute of Genomics (BIG), Chinese Academy of Sciences, under accession numbers HRA000155 that are publicly accessible at https://ngdc.cncb.ac.cn/gsa-human/browse/HRA000155.

Field-specific reporting

Please select the one below that is the best fit for your research. If you are not sure, read the appropriate sections before making your selection.

☒ Life sciences ☐ Behavioural & social sciences ☐ Ecological, evolutionary & environmental sciences

For a reference copy of the document with all sections, see nature.com/documents/nr-reporting-summary-flat.pdf

Life sciences study design

All studies must disclose on these points even when the disclosure is negative.

Sample size	No standard methods were used to predetermine sample size. We recruited patients who fulfilled the 2010 ACR/EULAR Rheumatoid Arthritis classification criteria and who underwent arthroplasty. The sample sizes are sufficient to provide stable single cell clustering results and to perform statistical analysis.
Data exclusions	No exclusion was applied to the uploaded raw data in ArrayExpress. For the final count matrix, we excluded cells based on pre-established criteria for single-cells: we excluded low quality samples and contaminating cells (i.e. - cells with low number of detected genes and high mitochondria content)-exclusion criteria for each case are comprehensively detailed in the relevant Methods section.
Replication	No experimental replication were performed in this study due to the nature of the study design.
Randomization	No randomization was performed due to the cross-sectional nature of the study.
Blinding	No blinding was performed in this study due to the cross-sectional nature of the study.

Reporting for specific materials, systems and methods

We require information from authors about some types of materials, experimental systems and methods used in many studies. Here, indicate whether each material, system or method listed is relevant to your study. If you are not sure if a list item applies to your research, read the appropriate section before selecting a response.

Materials & experimental systems

n/a	Involved in the study	
☐	☒	Antibodies
☒	☐	Eukaryotic cell lines
☒	☐	Palaeontology and archaeology
☒	☐	Animals and other organisms
☐	☒	Human research participants
☒	☐	Clinical data
☒	☐	Dual use research of concern

Methods

n/a	Involved in the study	
☒	☐	ChIP-seq
☒	☐	Flow cytometry
☒	☐	MRI-based neuroimaging

Antibodies

Antibodies used	magnetic beads:<br>Human CD45 Microbeads (Miltenyi Biotech, 130-045-801)<br>IHC antibodies:<br>Rabbit Anti-MCP4 antibody (Abcam, ab224593); Rabbit Anti-CCL18 (Abcam, ab233099); Rabbit Anti-CCL3 (Abcam, ab32609)
Validation	All the antibodies used in this study were commercial antibodies and were only used for applications, with validation procedures described on the following sites of the manufactures: https://www.miltenyibiotec.com/SG-en/products/cd45-microbeads-human.html#130-045-801; https://www.abcam.cn/mcp4-antibody-ab224593.html; https://www.abcam.cn/ccl18-antibody-ab233099.html; https://www.abcam.cn/macrophage-inflammatory-protein-1-alpha-ccl3-antibody-ab32609.html.

Human research participants

Policy information about studies involving human research participants

Population characteristics	RA-01: Female, 55~65Y, ACPA >3200; RA-02: Female, 40~50Y, ACPA negative; RA-03: Female, 60~70Y, ACPA 279;RA-04: Female, 30~32Y, ACPA >3200; RA-05: Female, 40~50Y, ACPA 332; RA-06: Female, 60~70Y, ACPA 35;RA-08: Female, 60~70Y, ACPA 588; RA-09: Female, 40~50Y, ACPA negative; RA-10: Female, 50~60Y, ACPA negative;RA-11: Female, 70~80Y, ACPA 329; RA-12: Female, 45~55Y, ACPA negative; RA-13: Female, 25~35Y, ACPA negative;RA-14: Female, 50~60Y, ACPA negative; RA-15: Female, 50~60Y, ACPA negative; RA-16: Female, 50~60Y, ACPA 55; RA-17: Female, 20~30Y, ACPA 114; RA-18: Female,

	50~60Y, ACPA 113; RA-19: Female, 40~50Y, ACPA negative; RA-20: Female, 20~30Y, ACPA negative; RA-21: Female, 40~50Y, ACPA negative.
Recruitment	Patients who fulfilled the 2010 ACR/EULAR Rheumatoid Arthritis classification criteria and who underwent arthroplasty were recruited in our present study. The patients were selected if they were clinically active and not taking disease-modifying antirheumatic drugs (DMARDS), corticosteroids or targeted therapies at the time of sampling.
Ethics oversight	Informed consent was obtained from all human participants, and our study was approved by the Peking Union Medical College Hospital Ethics Committee (no. JS-1940).

Note that full information on the approval of the study protocol must also be provided in the manuscript.
